# Ultrasound Therapy: Experiences and Perspectives for Regenerative Medicine

**DOI:** 10.3390/genes11091086

**Published:** 2020-09-17

**Authors:** Beatriz de Lucas, Laura M. Pérez, Aurora Bernal, Beatriz G. Gálvez

**Affiliations:** 1 Faculty of Biomedical and Health Sciences, Universidad Europea de Madrid, 28670 Madrid, Spain; beatriz.delucas@universidadeuropea.es (B.d.L.); laura.mp.hospital@gmail.com (L.M.P.); 2 Centro Nacional de Investigaciones Cardiovasculares (CNIC), 28029 Madrid, Spain; aurora.bernal@cnic.es

**Keywords:** ultrasound, LIUS, regenerative medicine, stem cells, proliferation, differentiation, migration, clinical trials

## Abstract

Ultrasound has emerged as a novel tool for clinical applications, particularly in the context of regenerative medicine. Due to its unique physico-mechanical properties, low-intensity ultrasound (LIUS) has been approved for accelerated fracture healing and for the treatment of established non-union, but its utility has extended beyond tissue engineering to other fields, including cell regeneration. Cells and tissues respond to acoustic ultrasound by switching on genetic repair circuits, triggering a cascade of molecular signals that promote cell proliferation, adhesion, migration, differentiation, and extracellular matrix production. LIUS also induces angiogenesis and tissue regeneration and has anti-inflammatory and anti-degenerative effects. Accordingly, the potential application of ultrasound for tissue repair/regeneration has been tested in several studies as a stand-alone treatment and, more recently, as an adjunct to cell-based therapies. For example, ultrasound has been proposed to improve stem cell homing to target tissues due to its ability to create a transitional and local gradient of cytokines and chemokines. In this review, we provide an overview of the many applications of ultrasound in clinical medicine, with a focus on its value as an adjunct to cell-based interventions. Finally, we discuss the various preclinical and clinical studies that have investigated the potential of ultrasound for regenerative medicine.

## 1. Introduction

Cell-based therapies that exploit our ever-expanding knowledge of disease processes and stem cell biology have the potential to address and improve a great number of conditions for which no cure or effective treatment is currently available. In the context of stem cell-based treatments, unfortunately, the majority of clinical trials to date show only modest functional improvements in damaged tissues. There are likely many reasons for this, including the difficulty in selecting the optimal cell population and the challenges in guiding the transplanted cells to the target tissue and ensuring their subsequent survival such that they can exert their regenerative actions [1,2]. Driven by these constraints, a number of strategies are being tested to improve the delivery/homing process of stem cells to target tissues, and to enhance engraftment, cell viability and regenerative capabilities [1]. Along this line, a novel strategy that has great potential to improve both homing and the regenerative capacity of stem cells is the use of ultrasound.

Ultrasound is defined as mechanical acoustic waves having a frequency above the upper limit of human hearing. Ultrasound technology has been used for more than fifty years in clinical practice, principally as a safe and non-invasive diagnostic tool, which currently allows for their use as a combined method for drug and gene delivery and therapeutic monitoring [3]. It has become an established and widely used intervention in biomedicine. Therapeutic ultrasound was born in the field of physical therapy, and comprises a group of heterogeneous ultrasound modalities that can be generally classed as being of “high-” or “low-intensity”, depending on whether their objective is to destroy tissue (e.g., kidney stone ablation) or to stimulate physiological processes (e.g., bone fracture repair), respectively [4]. Extracorporeal high-intensity focused ultrasound (HIFU) is an encouraging method for the non-invasive thermal or mechanical ablation of benign and malignant tissue [5]. Low-intensity pulsed ultrasound (LIPUS) was approved by the US Food and Drug Administration in 1994 for the accelerated healing of fresh fractures, and was soon thereafter approved for the treatment of established non-union [6]. A variation of LIPUS is the administration of ultrasound continuously rather than pulsed (100% duty cycle or pulsed ratio), which is known as continuous low-intensity ultrasound (cLIUS). In this context, low-intensity ultrasound (LIUS) would include both pulsed and continuous ultrasound. The success of LIUS in the field of bone regeneration has led to the development of new applications in the regeneration of soft tissue, including cartilage, tendons, and ligaments. Indeed, tissues/organs and also cells can respond positively to LIUS stimulation, resulting in improvements in cell properties and tissue regenerative capacities. Furthermore, LIUS can be used as an adjunct treatment to mesenchymal stem/stromal cell (MSC)-based therapies to bolster repair/regeneration [7,8]. Another variation of therapeutic ultrasound that has been tested to improve the success of cell therapy is pulsed focused ultrasound (pFUS), which is characterized by high-intensity localized pulses for short times to avoid harming the target tissue [9]. This modality has been shown to improve stem cell homing to target tissues [10], likely through its ability to locally upregulate cytokines and chemokines and create a chemotactic gradient for cell migration. Finally, while not considered conventional ultrasound, shock waves are acoustic pressure waves with the capacity to deliver mechanical forces to tissues, and are approved for kidney stone lithotripsy and for physical therapy, typically musculoskeletal applications [11,12]. Table 1 describes the various ultrasound modalities currently in use. Briefly, physical parameters of ultrasound comprise frequency, intensity, duty cycle, and pulse repetition frequency (number of pulses transmitted per second). Frequency is the number of times per second that a particle completes a compression and rarefaction cycle. Intensity is the time-average power transfer per area given in watts per square centimeter (W/cm^2^) and include the measure of spatial and temporal intensity, spatial average-temporal average (SATA). Duty cycle represents the ratio of time that the transducer is “on”.

The aim of this review is to describe the potential clinical applications of this emerging technology in the context of cell and tissue repair, and the status of clinical trials using ultrasound for regenerative medicine. 

## 2. Biological Effects of Ultrasound

A wide variety of cell types, including osteoblasts [13,14,15], chondroblasts [16], periodontal ligament fibroblasts [17,18,19,20,21], as well as MSC, induced pluripotent stem cell (iPSC), embryonic stem cell (ESC) [22,23], and endothelial cells [24], have been explored for their response to LIUS [25]. The ultrasound-induced changes observed in cells in vitro have also been examined in vivo.

LIUS exerts diverse effects in different tissues. In general, LIUS induces mechanical stimulation enhancing proliferation, differentiation, and maturation of many cell types, and stimulates the specific cell differentiation of MSC [26], improving osteoblast maturation. Interestingly, LIUS has also been reported to increase the viability, proliferation [27], and differentiation of stem cells [28], having a double-edged effect that may promote cell differentiation or the maintenance of stemness.

### 2.1. Proliferation and Viability

The ability of LIUS to induce proliferation in a broad range of cell types is well recognized. LIPUS stimulates the proliferation of adult stem cells in vitro. For example, human hematopoietic stem cells (HSC) show enhanced proliferation following LIPUS stimulationand maintain the expression of the progenitor cell markers CD34 and CD14 [28]. Similarly, LIUS boosts cell proliferation and colony-forming efficiency of human MSC while preserving their multipotency and karyotype. Mechanistically, it has been proposed that ultrasound upregulates cyclins, which modulate the cell cycle through the activation of extracellular signal-regulated kinase/mitogen-activated protein kinase (ERK/MAPK) and phosphatidylinositol 3-kinase/protein kinase B (PI3K/Akt) pathways [29,30,31]. Additionally, LIPUS was shown to promote fibroblast proliferation via activation of integrin receptors and the Rho/ROCK/ERK signaling pathway [32]. LIPUS also stimulates the proliferation of induced pluripotent stem cell-derived neural crest stem cells (iPSC–NCSC) [33]. The proliferative capacity of other cell types, including osteoblasts [34] and epithelial cells [35], is also enhanced by LIUS, indicating that this is likely a general phenomenon. 

Several studies have reported that LIPUS stimulates cell proliferation without impacting on cell viability [27,28] or apoptosis [36], establishing it as a safe tool to improve the properties of stem cells. Indeed, some studies have reported that ultrasound also enhances cell viability, as shown for human alveolar bone-derived MSC [37] and iPSC–NCSC [33]. Likewise, cLIUS seems to have a protective effect on MSC during chondrogenic differentiation, as revealed by an increase in cell viability in parallel with a decrease in apoptosis in three-dimensional (3D) alginate cultures. In this setting, cLIUS was shown to upregulate anti-apoptotic genes including Bcl-2 (B-cell lymphoma 2) and PCNA (proliferating cell nuclear antigen), which counteracted the induction of proapoptotic genes including P53 and Bax (Bcl-2 associated X-protein) stimulated by the addition of transforming growth factor beta 1 (TGβ-1) and 3D culture [38,39]. However, it has also been described that LIUS can induce apoptosis under specific conditions (usually higher intensities) and in specific cell types. These latter findings make LIUS a potential treatment for some cancers [40,41]. 

### 2.2. Adhesion

As mentioned, LIPUS stimulation increases the abundance of cell adhesion-related proteins such as integrins [42], fibronectin, and paxillin, and induces the formation of focal adhesions in MSC. Accordingly, LIUS could trigger the activation of cell adhesion processes through integrin signaling [43,44]. 

### 2.3. Extracellular Matrix Production

Largely related to the processes of chondrogenic and osteogenic differentiation, LIUS stimulation can induce the expression of extracellular matrix proteins not only in stem cells, but also in other cell types such as chondrocytes.

LIPUS-stimulated MSC show an increase in hydroxyapatite content and in the release of type I collagen and osteopontin, and this seems to be additive to the use of osteogenic differentiation medium [45]. Human nucleus pulposus cells are a promising cell therapy source for intervertebral disc degeneration, and LIPUS promotes the production of type II collagen, aggrecan, and Sox9 in these cells in vitro. In addition, LIPUS increases the secretion of TIMP-1 (tissue inhibitor of metalloproteinases 1) and reduces MMP-3 (matrix metalloproteinase-3) expression, which represents a positive balance for extracellular matrix (ECM) production. Mechanistically, this seems to involve the activation of the FAK/PI3K/Akt signaling pathway [46]. Similarly, LIUS stimulation increases the production of type II collagen and integrin β1 and reduces MMP-13 expression in rabbit chondrocytes via the integrin/p38 MAPK signaling pathway [47]. In human chondrocytes (C-28/I2 cell line), LIUS was found to induce the phosphorylation of other MAPK, JNK, and ERK signal transducers. The mechanotransduction pathways activated by LIUS seem to involve integrins and stretch-activated channels, and also MAPKs (JNK and ERK pathways) [48]. Thus, LIUS stimulation triggers integrins to mediate mechanotransduction and associated biochemical signaling through the cell membrane and downstream pathways, such as MAPK and PI3K/Akt [47].

### 2.4. Migration

MSC migration in vitro can be enhanced by LIPUS, and this phenomenon has been associated with increases in CXCR4, integrin-1β [49] and CCR-2 [42] expression, and also with cytoskeletal rearrangements involving the focal adhesion kinase (FAK)-ERK1/2 signaling pathway [50]. LIPUS treatment increases the migration of periodontal ligament stem cells (PDLSC) concomitant with an upregulation of TWIST1 and SDF-1, suggesting the involvement of the SDF1/CXCR4 signaling axis [51]. 

LIUS promotes the migration of other cell types such as osteoblasts [34], epithelial cells, and keratinocytes. In the latter example, LIUS-induced migration occurs through activation of the PI3K/AKT and MAPK c-Jun N-terminal kinase (JNK) pathways [52], whereas LIUS-induced migration of epithelial cells is associated with increased expression levels of adhesion-related genes, including integrin [35].

LIPUS stimulation of melanoma cells induces Rac activation through integrin-mediated cell-matrix adhesions, which promotes cell migration. Motility mechanisms stimulated by LIUS are dependent on the mechanosensitive focal adhesion protein vinculin and both FAK and Rab5 [53].

### 2.5. Homing

Ultrasound has great potential to enhance the recruitment and migration of stem cells to target tissue [50]. In the context of bone fractures, LIPUS induced the recruitment of MSC to the injury site, which resulted in improved callus formation and microarchitecture likely mediated by SDF-1 [54]. 

Pulsed focused ultrasound (pFUS) is an ultrasound modality that uses higher intensities and shorter times to stimulate tissues and generate a transitional gradient through the local upregulation of cytokines and chemokines, facilitating stem cell homing. Burks et al. studied the mechanism of action of pFUS in mouse hamstring muscles, finding an upregulation of cytokines, growth factors, and cell adhesion molecules without changes to the integrity of the tissue [55]. These molecules included SDF-1α, IL-1α, IL-1β, MCP-1, IFNγ, MIP-1α, GM-CSF, VEGF, FGF, HGF, PLGF, ICAM-1, and VCAM-1. The created cytokine gradient improved MSC homing and retention in the muscle [55]. In a mouse model of critical limb ischemia, the combination of pFUS and MSC had a superior therapeutic efficacy to the use of MSC alone. Specifically, pFUS stimulation increased MSC homing four-fold, resulting in an increase in vascular density, a reduction of the fibrotic area, and induced the expression of VEGF and IL-10 by MSCs [56]. 

### 2.6. Differentation

One of the most relevant features of LIPUS is its ability to stimulate cell differentiation. LIPUS can promote and accelerate cell differentiation and function in several ways, including increasing signal transduction and the synthesis of growth factors, inducing gene expression, stimulating enzymes in response to heat energy, enhancing metabolism, bolstering the number of blood vessels at the fracture site, enhancing mineralization and maturation, and inhibiting apoptosis and autophagy [57,58,59,60].

Interestingly, in the context of adult stem cells, LIPUS-mediated stimulation of HSC was found to enhance burst-forming unit-erythroid colony formation (of early erythroid progenitors) [28]. However, with respect to potential clinical applications, the majority of differentiation studies with LIUS have been performed with MSC or their derivatives under specific conditions of differentiation. For MSC cultured in the specific induction medium, LIPUS enhances the expression of osteogenic [31,37,45,59,61,62,63,64], chondrogenic [64,65,66,67,68,69,70] and adipogenic [59,71] markers, and also hepatic [72] and neural [36] lineages and astrocytes [73].

For instance, Cui et al. reported the beneficial effects of LIPUS on chondrogenic differentiation in bone marrow-derived MSC [74]. Besides, LIPUS enhanced TGFβ1-mediated chondrocyte differentiation of MSC by increasing the expression of chondrogenic genes and extracellular matrix proteins (type II collagen, aggrecan and SRY-related high mobility group-box gene 9 [SOX9] genes) [69,70], likely involving the integrin-mTOR signaling pathway [75] and the inhibition of autophagy [76]. 

In addition, in the presence of osteogenic differentiation medium, LIPUS enhanced the expression of osteogenic markers (type I collagen, runt-related transcription factor 2, osteopontin, osteocalcin, and heat shock proteins 70 and 90) in MSC [37,45]. The enhancement of osteogenic differentiation by LIPUS could be explained by the activation of the bone morphogenic protein (BMP) signaling pathway and the upregulation of heat shock proteins [77]. LIPUS also induces in vitro differentiation of other progenitor cells including osteoblasts [78].

It is evident that once MSCs are directed towards a certain fate, with the use of defined medium, LIPUS seems to enhance the differentiation towards that lineage. Interestingly, cells in soft and typically non-proliferating or low-proliferating tissues, such as cardiac and neural tissue, also respond to LIPUS. 

LIPUS stimulation also triggers cardiac-related gene and protein expression and the production of binucleated cells in cardiac mesangioblasts by mimicking the effects of 5-azacytidne, a well-known inducer of cardiac differentiation [49]. LIUS treatment improves cardiomyocyte differentiation in murine embryonic stem cells (ESC) by reducing spontaneous differentiation. LIPUS stimulation of ESC-derived embryoid bodies induces cardiac gene expression (cardiac troponin T and α and β myosin heavy chains) and enhances the beating rates of putative cardiomyocytes [22]. 

Besides, differentiation of MSC to the neuronal lineage is also enhanced by LIPUS through the induction of neural markers and neurotrophic factors [36,79]. With regards to pluripotent stem cells, LIPUS induces neural differentiation in vitro in iPSC-NCSC [33,80], which has the potential for the regeneration of injured nerves. In this case, FAK-ERK1/2 was the proposed signaling pathway for the induction of proliferation and differentiation of iPSC-NCSC [81]. Yang et al. evaluated the effects of LIPUS on iPSC-NCSC and found beneficial effects in the context of cell viability, proliferation, and neural differentiation for peripheral nerve tissue engineering [33]. 

Finally, LIPUS also plays a role on cell stemness. Some authors including Kusuyama et al. propose a role for LIPUS in stemness maintenance [82], highlighting again the double-edged effect that LIPUS can induce, likely depending on the cell type and the pathophysiological context of the tissue.

### 2.7. Regenerative Effects

The ability of LIPUS to stimulate cell differentiation and accelerate tissue repair has been extensively exploited in the tissue-engineering field. Tissue engineering encounters many challenges, especially with regards to restoring adequate mechanical strength, which is correlated with matrix production by the engineered tissues. In a recent study using a rabbit spinal cord injury model, LIPUS was found to enhance cell expansion, differentiation, and matrix production by different cells [36]. Likewise, LIPUS stimulation led to better structural formation in the context of new osteogenic and chondrogenic tissue formation, and integration of the newly formed tissues and original bone [36].

Bone growth and repair are under the control of biochemical and mechanical signals. LIPUS stimulation seems not only useful for long bones, but may also be used to accelerate bone regeneration of non-critical calvaria defects, as confirmed in a study with rats [83]. Similarly, a study performed in beagles showed that periodontitis might be ameliorated by LIPUS, as revealed by the acceleration of new alveolar bone formation, with a prospective for promoting periodontal tissue repair [84].

Several studies have also explored the combination of LIPUS and MSC infusion [7]. In rat bone defect models, co-treatment with MSCs and LIPUS was found to improve fracture healing for delayed union or non-union [8], and to enhance cartilage repair and subchondral reconstitution [85]. LIPUS was also shown to enhance spinal fusion in a rabbit posterior spinal fusion model using MSC-derived osteogenic cells [86]. Similarly, the combination of LIPUS and iPCS-NCSC administration was shown to promote the regeneration and reconstruction of rat transected sciatic nerves [87] and was beneficial for nerve injury-induced erectile dysfunction [88], pointing to LIPUS as a regenerative tool in the context of peripheral nerve regeneration.

### 2.8. Angiogenic Effects

Moreover, LIPUS increases angiogenesis and local blood perfusion [89]. The therapeutic angiogenic effects of LIPUS have been reported in endothelial cells, chick chorioallantoic membrane, and in a rat hind limb ischemia model [90,91,92,93]. Also, a very recent study combined LIPUS and collagen and collagen/hyaluronan scaffolds to enhance angiogenesis in cocultures of human adipose derived stem cells and endothelial cells [94].

LIPUS therapy triggers multiple angiogenic pathways, and has been shown to effectively increase capillary density and regional myocardial blood flow, and normalize ischemia-induced myocardial dysfunction without any adverse effects [95]. Interestingly, the aforementioned study showed that LIPUS therapy ameliorates post-myocardial infarction left ventricular remodeling in mice, with pivotal roles played by the mechanotransduction system, including β1-integrin and caveolin-1 and associated downstream pathways [89]. A β-1integrin signaling pathway was also proposed by Bernal et al. to explain the mechanotransduction induced by LIPUS on cardiac mesoangioblasts and the enhancement of cardiac lineage differentiation [49].

### 2.9. Anti-Inflammatory Effects

LIPUS has been recently reported to have biologic relevance in the context of inflammation. LIPUS treatment was shown to alleviate the lipopolysaccharide-induced inflammatory response in murine RAW264.7 macrophages by increasing the activation of caveolin-1 [95], and also improved the general status of coxsackievirus-B3-infected mice including their ventricular function by suppressing CD68+ infiltration in the heart [96,97]. Related to inflammation, LIPUS was found to inhibit the inflammatory pathways activated during aseptic joint loosening [97], suggesting an anti-inflammatory role for LIPUS in the orthopedic context. In a similar line, LIPUS was shown to inhibit the production of mature IL1β in a murine model of synovial inflammation [98], and to reduce histological damage and lesion size in the joints of mice with systemic autoimmunity [99]. These studies, overall, suggest an important role of LIPUS not only as a regenerative stimulator but also as an anti-inflammatory treatment, and constitute the basis for a new therapeutic strategy for joint inflammatory diseases. Indeed, LIPUS is suggested to act on both anti-inflammatory and cell differentiation pathways to enhance the outcome of tissue engineering treatments of periodontitis by suppressing inflammation and increasing osteogenic differentiation [100]. Moreover, LIPUS has been proposed as a treatment option for autoimmune diseases [101].

### 2.10. Anti-Degenerative Effects

LIPUS might also function to protect against degenerative processes in neural tissue. It has been shown LIPUS stimulation increases the protein levels of BDNF, GDNF, VEGF, and GLUT1 in rat brain astrocytes, which have an important role in the growth and survival of developing neurons by secreting neurotrophic factors [102]. In this case, an integrin inhibitor was found to attenuate LIPUS-induced neurotrophic factor expression, suggesting that neurotrophic factor protein levels may be promoted by LIPUS through integrin receptor signaling, consistent with the model proposed in the cardiac context [49]. In addition, LIPUS stimulation protected cells against aluminum toxicity and reduced cerebral damage in terms of myelin loss and apoptosis [102]. Considering that some of these factors have emerged as major molecular players in the regulation of neural circuit development and function, LIPUS may play a beneficial role in neurodegenerative diseases [73].

Regarding the central nervous system, it has been shown that LIPUS protects retinal ganglion cells from apoptosis [103]. Application of LIPUS following nerve surgery may promote nerve regeneration and improve functional outcomes through a variety of mechanisms, including an increase of neurotrophic factors, Schwann cell activation, cellular signaling activation, and induction of mitosis [88].

The most frequently used ultrasound parameters to improve the properties of cells and the regenerative capacity of tissues are shown in Table 2.

## 3. Mechanism of Action

Cell behavior (quiescence, proliferation, migration, differentiation…) can be regulated by chemical (cytokines, hormones, growth factors...) and physical (components of the extracellular matrix, mechanical stimuli, forces...) factors, and also by their relationship with other cells. Belonging to the physical factors, ultrasound is a mechanical signal that can be sensed by stem cells and other cell types such as chondrocytes, epithelial cells, and osteoblasts [29,78]. Ultrasound are able to induce biological effects trough thermal and non-thermal mechanism as mechanical stress, cavitation or gas body activation. LIUS has demonstrated the potential to exert physical force on cells [104]. However, the biophysical mechanisms for their therapeutic action remains unknown [4]. Is better known their safety. The use of low intensity ultrasound induce negligible thermal effects on cells or on living tissues, and there is no microbubbles formation or cavitation [105,106]. The activation of cell mechanoreceptors by ultrasound translates mechanical signals into biochemical responses in the cell resulting in the modulation of biological events linked to survival and growth, among others [107].

**Figure 1:**
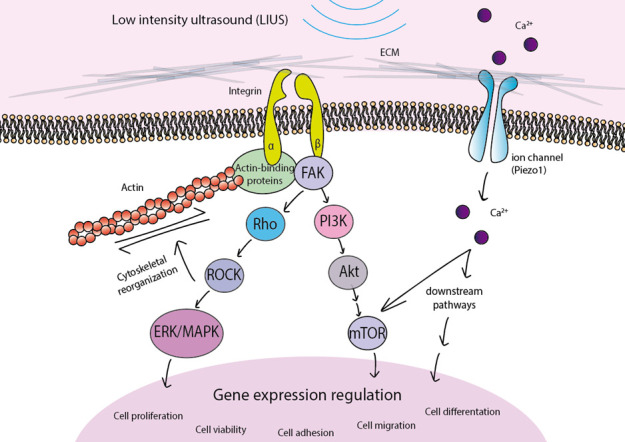
Stimulation of cells by low-intensity ultrasound activates mechanoreceptors including integrins and Piezo1. The subsequent phosphorylation of signaling proteins activates diverse pathways such as Rho/ROCK/ERK/MAPK and PI3K/Akt/mTOR, which ultimately govern different cellular responses including cell proliferation, viability, adhesion, migration or differentiation.

The above examples serve to illustrate that the different in vitro LIUS stimulation methods in several cell models seem to converge on similar and shared signaling pathways (Figure 1). Cells “sense” ultrasound mechanical stimulation through transmembrane proteins such as integrins and ion channels [48,108]. Mechanotransduction processes sense environmental mechanical signals and translate them into biochemical responses of the cells. This mechanism requires cell–matrix or cell–cell adhesion plus contractility of the actin cytoskeleton to sense and respond to changes. Integrins act as physical linkages of the cell actin cytoskeleton with the ECM, and mediate attachment and migration of cells [107]. The activation of integrins triggers the recruitment of different cytoskeletal adaptor proteins (talin, vinculin, paxillin…) that bind to the actin cytoskeleton, resulting in the clustering of focal adhesions [109]. LIUS stimulation activates integrins, inducing the formation of stress fibers and focal adhesions. This engagement stimulates signaling cascades that converge on the Rho family of small GTPases, which are powerful modulators of actin cytoskeletal rearrangements and cell migration. Briefly, RhoA activates Rho kinase (ROCK), which in turn promotes myosin II activity [49,107]. Downstream of Rho/ROCK, LIUS stimulates ERK/MAPK signaling. Focal adhesion assembly can, in turn, activate other downstream signaling pathways, including PI3K/Akt [110]. 

In addition to integrins, other proteins have been described as mechanoreceptors for LIUS stimulation. A study performed on chondrocytes proposed that LIUS stimulation might be mediated via stretch-activated ion channels and integrins and, subsequently, through JNK and ERK pathway activation [48]. More recently, Piezo mechanosensitive ion channels have been implicated in transducing the LIPUS response in cells [106,111,112], with Piezo1 proposed as an important regulator of MSC fate by determining BMP2 expression [113] and differentiation potential. It has also been described that Piezo1 can increase Ca^2+^ and activate Akt/mammalian target of rapamycin (mTOR) signaling pathways [114].

Cells use mechanoreceptors to sense LIUS, resulting in the activation of important downstream signaling cascades that regulate gene expression to affect fundamental cell functions including cell proliferation, cell viability, adhesion, migration, and differentiation. 

## 4. Therapeutic Applications of Ultrasound 

Diverse ultrasound procedures have been developed in the last decades to induce tissue regeneration. These procedures can be performed using a cell-based strategy with different cell types (e.g., MSC, progenitor cells…) or cell-free (e.g., physical or chemical stimulators, viral or non-viral vectors…). However, as discussed earlier, there is evidence that a combination of different strategies has the highest beneficial potential. A range of ultrasound-based interventions has been successfully tested in preclinical studies, and some of them have advanced to clinical trials with humans.

### 4.1. Search Strategy

We performed a systematic literature review of clinicaltrials.gov and PubMed. Selected studies met the following inclusion criteria: (1) Preclinical studies carried out in animals using diverse ultrasound procedures; (2) Clinical trial studies in any phase of clinical development, but especially in phase II/III testing therapeutic ultrasound efficacy; (3) The study design has a control group and one experimental arm using therapeutic ultrasound; (4) The primary outcome for the preclinical or clinical studies selected was increased tissue healing or improved disease control; (5) The studies selected are original research articles and had appropriate Institutional Review Board approval and informed consent procedures for humans or appropriate local Institutional Care and Animal Use Committee approval for animals. (See Supplementary File S1: Database files for complete data literature generated).

### 4.2. Preclinical Studies with Ultrasound 

Preclinical studies are necessary to assess the safety and efficacy of ultrasound, and to validate the positive effects of therapies for the promotion of healing. Diverse approaches have been used to explore this, including in vitro studies and animal models, and to better understand the pathways involved in regeneration. The bulk of preclinical studies have assessed bone or cartilage regeneration, and relatively few studies have examined tissue remodeling in other systems or pathologies, including muscular or cardiovascular pathologies.

#### 4.2.1. Preclinical Studies in Bone Healing

The search with the possible combinations of keywords; "LIPUS", “animal model” and “bone regeneration"; generated 119 entries in PubMed (June 2020), of which 58 are associated with the use of LIPUS in bone remodeling, 7 are reviews and the remainder use shock waves to stimulate bone healing (Table 3). 

The first preclinical studies of LIPUS in animal models were published in the 1990s, showing that LIPUS applied for 20 minutes daily was useful for bone repair and new bone formation in rabbits [115]. Other studies focused on treatment combinations and demonstrated that fracture healing can be enhanced by combining MSC with LIPUS, which promotes new bone formation and accelerates bone remodeling in rat models [116]. During the next decade, several other preclinical models were used such as mouse, rat, or rabbit, and these studies increased in number in the ‘10s (i.e., 2010–2020). 

#### 4.2.2. Preclinical Studies in Other Tissues

Beyond bone repair, other studies on tissue regeneration focused mainly on muscular, nerve, and dental regeneration. A search with the possible combinations of keywords "LIPUS", “animal model” and different “tissue regeneration" options generated 37 entries in PubMed (June 2020), and 19 of these articles described shock waves as ultrasound therapy (Table 4).

Focusing on LIPUS therapy, six studies examined muscular regeneration, mainly in rats. The most recent work was published in 2019, and investigated the therapeutic effect of LIPUS in a urinary incontinence rat model [117]. The authors found that LIPUS restored bladder capacity and activated satellite cell myodifferentiation. Five studies assessed nerve regeneration in rats. Sato et al. (2016) described a possible novel therapy for inferior alveolar nerve injury, frequently caused by trauma or surgery, using a daily treatment LIPUS protocol [118]. More recently, Xia et al. (2019) described the effects of LIPUS combined with iPSC-NCSC, perfluorotributylamine, and growth differentiation factor 5 for the repair of peripheral nerve injury [81]. Five studies were found on dental regeneration, describing a regenerative effect of LIPUS to repair dentin-pulp complex injury in rats [119]. Finally, one study evaluated the use of LIPUS in wound healing [120] or other tissue regeneration.

### 4.3. Clinical Trials with Ultrasound

Ultrasound was traditionally used in rehabilitation medicine as an imaging modality to assess the musculoskeletal system. As a therapy, numerous clinical trials have been performed during the last few years. A general search in clinicaltrials.gov using “ultrasound” and “regeneration” resulted in 52 matches, but only 6 of them are related to the use of ultrasound for tissue remodeling or healing (Table 5). All of them are interventional studies, with a randomized design and in a non-determined phase of this study.

The first clinical study with ultrasound was in 2014 for hypertension (NCT02042066). The study evaluated the safety and efficacy of shock waves for the treatment of resistant hypertension, and the results were unpublished. However, none of the clinical trials have published their results yet. 

A more specific search to select only “low-intensity pulse ultrasound” as therapeutic choice, resulted in 22 matches, of which 10 focused on bone healing (Table 6). The first clinical trial performed using LIPUS as a treatment for bone healing was published in 2007 (NCT00423956). The study enrolled 72 subjects randomized into two arms, and the procedure was to induce tooth root resorption by ultrasound or by sham control. The same group previously published a study with twelve female subjects where the use of LIPUS during orthodontic treatment enhanced the repair of root resorption [121]. They also observed an increase in pulp vascularity in the pulp, according to a published report on new blood-vessel formation by ultrasound [122].

Several other clinical trials have tested LIPUS to treat osteoarthritis, fractures, or bone degenerative diseases, with all examining tissue remodeling. Indeed, a clinical practice guideline has been recently published for the use of this therapy in bone healing [123]. One of the main clinical trials reporting on bone remodeling is the TRUST Study (NCT00667849), which is the only trial with results to date [124,125]. This trial enrolled 501 patients in a multi-center randomized study; patients were allocated to receive daily LIPUS or sham on their tibial fracture. The aim of the study was to evaluate healing, and the results were negative for the use of LIPUS to improve functional recovery. By contrast, Zhou et al. [126] published a meta-analysis on the use of LIPUS for knee osteoarthritis and found a beneficial effect of LIPUS on pain relief and functional knee recovery.

The latest development in LIPUS technology concerns the application of the therapy in a focused manner (LIFUP, “Low-Intensity Focus Ultrasound Pulsation” or FLIPUS, “Focused Low-Intensity Pulsed Ultrasound”). Clinical trials have tested the effect of this type of LIPUS in different therapeutic scenarios for example, to open the blood–brain barrier in patients for delivery of therapeutic compounds in cancer (NCT03744026, NCT04021420) or brain diseases (NCT03119961). Furthermore, there are clinical trials examining the effects of ultrasound on arterial endothelial function, implying beneficial anti-inflammatory and vasodilatory effects, for example in patients with diabetes (NCT02872922). The group involved in this study previously published results in healthy volunteers, and they found that continuous and pulsed therapeutic 1-MHz ultrasound waveforms improved endothelial function, providing anti-inflammatory vascular effects [127].

In summary, there are a number of varied ongoing clinical trials with different types of ultrasound that may offer a new therapeutic approach for many diseases as a main or adjuvant treatment to enhance cell therapy and/or promote tissue healing, for instance, directing MSC differentiation to osteoblasts for bone formation [116], stimulating dental regeneration [18], or even for treatment of spinal cord injuries [36]. Accordingly, LIPUS could significantly increase the level of stem cell differentiation [25] and could be used as a novel cell therapy strategy. However, many of the preclinical trials developed to date have not been tested on humans, so it remains unknown whether they will clinically effective.

## 5. Limitations

Finally, it is important to discuss the current limitations of LIPUS as a biostimulator in clinical therapy. It is quite striking that some studies support the use of LIPUS to differentiate MSC toward an osteogenic fate [64], whereas other studies report chondrogenic differentiation [68]. These discrepancies may arise because of the different LIPUS stimulation settings [9]. LIPUS is a physical phenomenon with some characteristic features within a specific range, and it will be necessary to establish exactly the type of stimulation that is performed. Indeed, intensity-related effects have been reported; for example, 30 and 90 mW/cm^2^ of LIPUS at frequencies of 1–3 MHz were the more effective for cellular differentiation responses and in vitro cell biostimulation [25]. These authors showed that 20, 30, 40, and 50 mW/cm^2^ were suitable for the differentiation of bone marrow-derived MSC, with these intensities promoting differentiation through the up-regulation of HSP70 and HSP90 expression, activation of the BMP signaling pathway, and inhibiting autophagy [76,77]. However, another study indicated that alkaline phosphatase activity, a marker of osteoblastic cell differentiation, was enhanced at 125 mW/cm^2^ during osteoblast differentiation [128]. 

Further studies are required to clarify these discrepancies and to determine the best parameters of LIPUS stimulation as a new therapy.

## 6. Conclusions

Although ultrasound has shown great potential for the treatment of different conditions, there is a critical need for further studies and the design of standardized protocols (with established parameters of use). This is fundamental for understanding the mechanisms involved for regenerative medicine. While ultrasound continues to be extensively studied, there remains a paucity of human studies. Clinical trials mainly concern the bone field and an improvement in therapeutic treatment of certain diseases. The extensive number of preclinical studies should lead to a better understanding of how ultrasound improves stem cell therapy for the treatment of different conditions or diseases.

## Figures and Tables

**Table 1:** Methodologies of therapeutic ultrasound and shock wave application.

		Parameters	Applications
Therapeutic Ultrasound (<5 MHz)	Traditional	0.1–3 W/cm^2^ SATA, frequency 1–3 MHz	tendonitis, osteoarthritis and pain relief
	LIPUS	30–100 mW/cm^2^ SATA, frequency 1.5 MHz, 1 kHz, duty cycle of 20%	Bone fracture healing, soft tissue regeneration, anti-inflammatory effects…
	cLIUS	30–100 mW/cm^2^ SATA, frequency 1.5 MHz, 1 kHz, duty cycle of 100%	
	pFUS	133 W/cm^2^ SATA, frequency 1 MHz, 5 Hz, duty cycle of 5%	Chemoattractant local and temporal gradient for homing process
	HIFU	400–10,000 W/cm^2^ SATA, frequency 0.8–4 MHz	non-invasive thermal or mechanical ablation of benign and malignant tissue
Shock waves		300–3000 pulses, energy 0.05–0.12 mJ/mm^2^	kidney stone lithotripsy, physical therapy

SATA: spatial average-temporal average; LIPUS: Low intense pulsed ultrasound; cLIUS: continuous low-intensity ultrasound; pFUS: pulsed focused ultrasound; HIFU: high-intensity focused ultrasound.

**Table 2:** More frequent ultrasound parameters used for regenerative effects. The most common ones are highlighted in bold.

Biological Effects	Ultrasound Parameters	References
Proliferation and viability	frequency 1/**1.5** MHz; **30**/50/100 mW/cm^2^ SATA; **20%**/100% duty cycle; 10/**20**/30 min/day	[28,30,32,33,34,36,39,41]
Adhesion	frequency **1.5** MHz; **30**/100 mW/cm^2^ SATA; **20%**/100% duty cycle; 10/**20** min/day	[42,43,44]
Extracellular matrix production	frequency 1/**1.5**/3 MHz; **30**/200 mW/cm^2^ SATA; **20%** duty cycle; 15/**20** min/day for 5/7 consecutive days	[45,46,47,48]
Migration	frequency 1/**1.5**/3 MHz; **30**/160/240 mW/cm^2^ SATA; **20%**/100% duty cycle; 15/**20** min/day	[34,35,42,51,53]
Homing	LIPUS: frequency 1.5 MHz; 30 mW/cm^2^ SATA; 20% duty cycle; 20 min/day for 3 days pFUS: 133 mW/cm^2^ SATA; frequency 1 MHz; 1/5 Hz; 5% duty cycle	[54,55,56]
Differentiation	frequency **1**/**1.5**/2 MHz; **30**/50/150/**200**/300/500 mW/cm^2^ SATA; **20%** duty cycle; 10/**20**/30 min/day	[28,36,45,62,64,67,72,77,81]
Regenerative effects	frequency 1.5/1.6 MHz; 30/50/90 mW/cm^2^ SATA; 20% duty cycle; 20 min/day	[8,36,83,84,86]
Angiogenic effects	frequency 1/1875 MHz; 15/25 mW/cm^2^ SATA; 20% duty cycle; 20 min/day	[89,91,94,95]
Anti-inflammatory effects	frequency **1.5**/3 MHz; **30**/200 mW/cm^2^ SATA; **20%** duty cycle; 15/20 min/day	[95,97,99,101]
Anti-degenerative effects	frequency 1 MHz; 50/110 mW/cm^2^ SATA; 20%/50% duty cycle; 10/15 min/day	[73,102,103]

**Table 3:** Preclinical studies carried out in different animal models for bone healing (LIPUS or shock wave treatment alone, or combined with other therapies, e.g., mesenchymal stem/stromal cell (MSC), grafts or chemical compounds).

	Mouse	Rat	Rabbit	Others	Total
**LIPUS**					
1990–1999	0	1	1	0	**2**
2000–2009	2	5	4	3	**14**
2010–2020	5	23	13	1	**42**
**Shock Wave**	-	-	-	-	**31**

**Table 4:** Preclinical studies carried out in different animal models for other tissue healing (LIPUS or shock wave treatment alone or combined with other therapies, e.g., MSC, grafts, or chemical compounds).

	Mouse	Rat	Rabbit	Other	Total
**LIPUS**					16
1990–1999	1	13	0	2	
**Shock Wave**	-	-	-	-	19

**Table 5:** Clinical studies carried out for tissue healing with different ultrasound devices.

NCT	Condition	Treatment	Outcome
NCT03705039	Knee osteoarthritis	Ultrasound (1 mW/cm^2^, frequency 1 MHz, 1 kHz, ratio 1:4, 10 min)	Synovial fluid and cartilage thickness
NCT03147313	Peripheral nerve injury	Shock wave (300 or 500 pulses, frequency 3 Hz, energy 0.1 mJ/mm^2^)	regeneration of peripheral nerve injuries
NCT04123782	Muscle injury	Focus Extracorporeal shock wave (3000 impulses at 0.12 mJ/mm^2^)	Muscle injuries recovery
NCT02042066	Hypertension	Shock wave (0.09–0.1 mJ/mm^2^)	Increase tissue perfusion
NCT02800200	Carpal tunnel syndrome	Plasma and Shock wave (100 MPa, 10 μs).	Regeneration of peripheral neuropathy
NCT03986359	Erectile dysfunction	Shock wave (0.05 mJ/mm^2^, 3000 pulses)	Increases erection hardness score

**Table 6:** Clinical studies carried out for tissue healing with different LIPUS therapies. (LIPUS parameters 30–100 mW/cm^2^, frequency 1.5 MHz, 1 kHz, duty cycle of 20%).

NCT Number	Conditions	Interventions	Outcome
NCT00423956	Root resorption	LIPUS	Induced inflammatory root resorption
NCT00744861	Lumbar degenerative disc disease	LIPUS	Posterolateral fusion success
NCT00931749	Knee osteoarthritis	LIPUS	Knee cartilage thickness and volume
NCT01623804	Knee osteoarthritis	LIPUS	Pain and physical function
NCT02253212	Glioblastoma	LIPUS + Drug	Blood–brain barrier opening
NCT02034409	Osteoarthritis	LIPUS	Symptoms reduction
NCT02383160	Fractures	LIPUS	Time to union of scaphoid non-unions
NCT00667849	Tibial fractures	LIPUS	Healing of tibial fractures
NCT02872922	Diabetes mellitus	LIPUS	Changes arterial endothelial function
NCT03251807	Malocclusion	LIPUS	Dentoskeletal changes
NCT03119961	Brain diseases	LIPUS	Blood–brain barrier opening
NCT03347084	Brain diseases	LIFUP	Improvements in cognitive functioning
NCT03329482	Low back pain	LIPUS	Pain intensity of patients
NCT03744026	Glioblastoma	LIPUS + Drug	Blood–brain barrier opening
NCT03679507	Osteoarthritis in the knee	LIPUS	Pain intensity measure
NCT03657056	Temporal lobe epilepsy	LIFUP	Changes of the blood-oxygenation level
NCT03717922	Brain diseases	LIFUP	Auditory verbal learning
NCT04021420	Metastatic melanoma	LIPUS + Drug	Blood–brain barrier opening
NCT04131387	Female stress urinary incontinence	LIPUS	Urinary incontinence questionaire
NCT03868293	Drug resistant epilepsy	FOCCUS LIPUS	Reducing seizure frequency
NCT04406337	Osteoarthritis	LIPUS	Visual analog scale (Pain)
NCT04339972	Healthy adults	LIFUP	Analgesia

LIFUP: Low-Intensity Focus Ultrasound Pulsation.

## Supplementary Materials

The following are available online at https://www.mdpi.com/2073-4425/11/9/1086/s1, File S1: Database files for complete data literature generated.
